# Evaluation of an Electronic Application for Enhancing Medication Adherence Among Hypertensive Patients: A Qualitative Research Based on Consolidated Framework for Implementation Research (CFIR)

**DOI:** 10.1002/hsr2.70758

**Published:** 2025-04-29

**Authors:** Sichen Wang, Junjie Huang, Apurva Sawhney, Xianjing Liu, Chaoying Zhong, Jianli Lin, Junjie Hang, Claire Chenwen Zhong, Jinqiu Yuan, Martin C. S. Wong

**Affiliations:** ^1^ The Jockey Club School of Public Health and Primary Care, Faculty of Medicine The Chinese University of Hong Kong Hong Kong SAR China; ^2^ Centre for Health Education and Health Promotion, Faculty of Medicine The Chinese University of Hong Kong Hong Kong SAR China; ^3^ Department of Radiology and Nuclear Medicine Erasmus MC University Medical Center Rotterdam the Netherlands; ^4^ Department of Electrical Engineering and Automation Guangdong Ocean University Guangdong China; ^5^ Peking‐Tsinghua Center for Life Sciences, Academy for Advanced Interdisciplinary Studies Peking University Beijing China; ^6^ Cancer Hospital and Shenzhen Hospital Chinese Academy of Medical Sciences and Peking Union Medical College Guangdong China; ^7^ Clinical Research Center, Big Data Center, The Seventh Affiliated Hospital Sun Yat‐Sen University Guangdong China

**Keywords:** adherence, eHealth, hypertension, patient adherence, patient education

## Abstract

**Background and Aims:**

Patient adherence plays a crucial role in effective chronic disease management. eHealth interventions utilizing smartphone applications (apps) are emerging as a promising approach to support adherence. This study aims to explore the acceptability, usability, perceived benefits, and barriers by app users, both with or without chronic diseases.

**Methods:**

In‐depth interviews were conducted with 12 Cantonese‐speaking patients aged 40 years or above who were eHealth app users. Participants' perceptions of the app, their preferences, and the impact on health outcomes were analyzed using the Consolidated Framework for Implementation Research (CFIR).

**Results:**

Participants had positive perceptions and understanding of the app, with a majority preferring “My eDrug Manager” over other similar apps. Monitoring medication and blood pressure improved user habits, while medication details and various reminder styles enhanced patient adherence. However, participants emphasized the need for efficient technical improvements and timely customer support. Some users found the app incompatible for disabled patients, while others desired comprehensive, customizable features adaptable to individual needs. Varying levels of interactivity required by users had the potential to negatively impact their perceptions of the app.

**Conclusion:**

This study provides insights into the benefits and concerns regarding user intention to adopt the eHealth app. Participants suggested improvements for different reminder styles and comprehensive health reports. Findings can inform policy makers about the current situation and how implementing user feedback could increase app usage, medication adherence, and ultimately improve individual health outcomes.

**Clinical Registration:** Not applicable.

## 1 Introduction

Patient adherence is crucial to assessing medication effectiveness and managing chronic diseases optimally. The World Health Organization (WHO) defines *adherence* as “the extent to which the patient follows medical instructions” [1]. Non‐adherence, conversely refers to intentional or unintentional deviations from prescribed treatments, manifesting as underutilization, overutilization, and incorrect medication consumption. Unintentional reasons include forgetfulness, limited knowledge about their medical conditions or prescribed medications, physical barriers, and complexity of treatment regimens [2]. Intentional reasons could stem from patient–provider communication, patient's level of agreement with the prescribed regime, or other perceptions and constraints. A recent WHO and National Institute for Health and Clinical Excellence investigation found non‐adherence rates of around 30% to 50% among chronic disease patients, presents significant challenges to global health [1].

Polypharmacy is the use of five or more medications, while hyper polypharmacy refers to the use of 10 or more chronic medications. Both are linked to increased non‐adherence risk. Seven drug classes frequently prescribed among the elderly (65–94 years old) were assessed for adherence using the medication possession ratio (MPR), which revealed a cumulative low adherence as well as across multiple drug categories [3]. Notably, with each additional drug prescribed, the cumulative adherence as well as adherence across all seven drug categories declined [3]. This issue is exacerbated by the growing aging population and healthcare pressures [3, 4].

Suboptimal adherence has been linked to suboptimal blood pressure control in hypertensive patients [5]. In 2014–2015, the prevalence of hypertension among the Hong Kong population aged over 15 years was found to be 27% [6], 64.8% of these subjects fell within the age range of 65–84 years [6]. Recognizing the need for physicians to implement effective hypertension management strategies, the Hong Kong Reference Framework for Hypertensive Care was created [7].

Innovative solutions are needed to address these issues. Mobile health (mHealth) interventions, such as smartphone applications (apps), have shown promise in supporting medication adherence for chronic diseases [8, 9]. According to a survey, 16% of patients relied on reminders to take their medication, and interestingly, two‐thirds of smartphone users habitually checked their devices for messages, alerts, or calls, even without notifications [2]. Electronic health (eHealth) devices similarly offer a cost‐effective alternative to traditional adherence interventions [8]. Evidence suggests that reminder systems such as text messaging and internet‐based interventions have demonstrated promising results, with one study reporting a striking improvement in adherence from 65% to 94% after implementing a telemedicine chronic disease management program [8, 10]. These findings underscore the potential of mHealth and eHealth interventions in improving medication adherence and patient care.

High rate of polypharmacy, potential adverse drug reactions, and low adherence among the elderly with multi‐morbidity strain the already overwhelmed Hong Kong public healthcare system. “My eDrug Manager” is the first integrated medication management app developed by pharmacists and available to the public in Hong Kong. It provides access to professionally written drug monographs, summarizing key drug information that has been shown to support patient awareness and compliance with the prescription. Several components of “My eDrug Manager” are inspired by evidence‐based chronic disease patient management widely used in Western countries [11]. However, “My eDrug Manager” and its novelty lies in creating an innovative combination of these varied management strategies into a single mobile app. The app aims to strengthen medication adherence among elderly hypertensive patients, although limited literature may impact the ability to evaluate the intervention effectively. This study aims to explore participants' perceptions and attitudes toward adopting electronic apps, informing future healthcare services.

## 2 Methods

### 2.1 Ethics Approval Statement

This study was approved by the Joint Chinese University of Hong Kong—New Territories East Cluster (CUHK‐NTEC) Clinical Research Ethics Committee; reference no. 2018.650, Hong Kong SAR. Before the interviews, participants signed written informed consent forms and completed an anonymous demographic survey. Moderators reassured participants about data confidentiality, secure storage of collected data, and the voluntary nature of their participation, emphasizing their right to withdraw at any stage of the study. The study also uses COREQ for qualitative research according to the EQUATOR Network (SRQR) [12].

### 2.2 Study Design and Participants

Participants were purposively sampled from January 2022 to August 2022, ultimately recruiting 12 participants from a large‐scale study recruited around 558 community‐dwelling elderly subjects and aimed to improve medication adherence among elderly hypertensive patients. All subjects were selected using the following criteria: aged 40 years or over, Cantonese‐speaking, prescribed at least one antihypertensive agent by physician at time of study, demonstrate poor medication adherence, possess a compatible smartphone and users of the eHealth and “My eDrug Manager”, living independently without assistance for medication reminders by caretakers, and absence of any medical concerns interfering with their ability to comprehend instructions and provide informed consent. “My eDrug Manager” is a free‐of‐charge, Hong Kong's first drug monitoring app developed by pharmacists, aiming to provide an integrated medication management, hence to assist further adherence to any prescribed therapies. “My eDrug Manager” was downloaded by a shared link to subjects to install via a one‐to‐one coaching, introduced and instructed subjects to use each function of the app, and ensured that they scheduled their medication correctly in the app. The poor medication adherence was defined by an MMAS‐8 score of ≤ 6. The Morisky Medication Adherence Scale (MMAS‐8) is a validated eight‐item questionnaire that assesses patients' self‐reported adherence to medication regimens. A score of ≤ 6 on the MMAS‐8 indicates poor adherence.

### 2.3 Data Collection

Focus groups were created by groups with varied participant characteristics, including age (40–64 vs. ≥ 65 years), gender, number of chronic medications taken (to determine polypharmacy) (Table S1), and educational level. Four groups were created, with at least three in‐person interviews conducted per group until data saturation was achieved. The maximum variation sampling strategy was employed to capture diverse perspectives. Interviews were conducted by an experienced moderator using a pre‐designed guide of open‐ended questions, while another researcher acted as an observer and took notes. The interviews were conducted via phone, audio‐recorded, and lasted approximately 30 –45 min. The themes emerged from an inductive analysis of interview transcripts. Data saturation was determined when no new themes emerged after three interviews and existing themes were repeatedly confirmed by participants.

The interview guide was based on the nine constructs of the CFIR, ensuring comprehensive data collection:

1. Intervention source: Background information that participants knew about the app at the interview stage.
2. Evidence strength and quality: Evidence which may affect participant attitudes and perceptions regarding the app.
3. Relative advantage: Participants' attitude and perception of the advantage and disadvantages of the app versus an alternative solution.
4. Adaptability: The degree to which the app can be adapted, tailored, refined, or reinvented to meet local needs.
5. Trialability: The extent to which the app is available for patients to use.
6. Complexity: Perceived difficulty in using the app.
7. Design quality and packaging: Perception in how the app is presented or instructed.
8. Cost: Various areas of the app that need funding.
9. Other comments on the use of the app.

### 2.4 Data Processing and Analysis

Recorded interviews were transcribed verbatim, then analyzed by NVivo 10 software (QSR international, Australia). Initially, an independent researcher performed coding based on CFIR domains, segmented into individual units followed by the identification and development of additional codes for every interview separately. Axial coding was utilized when multiple categories converged around a core phenomenon, resulting in the creation of a code book for each interview analysis. A refined thematic structure was achieved through consensus among the research team. The transcriptions were translated into English and independently reviewed by two researchers for completeness and accuracy. Data was organized by content and sorted into thematic chapters and sections for ease of analysis. Microsoft Word and Microsoft Excel were used for data consolidation and analysis. A sample size of 12 is justified in this qualitative study because it is adequate to reach data saturation, ensuring that no new themes emerge while allowing for in‐depth exploration of participants' experiences and perspectives; this size also balances the need for rich, detailed insights with practical constraints such as time and resources, and it aligns with precedents in similar qualitative research that have successfully utilized comparable sample sizes to yield meaningful findings. To strengthen the study's reliability and validity, we employed an audit trail that ensures transparency and reproducibility, peer debriefing that enhances objectivity by providing critical feedback from colleagues, and prolonged engagement that fosters deeper understanding and trust with participants.

## 3 Results

### 3.1 Theme 1: User Understanding of the Electronic App

Understanding of “My eDrug Manager” app by the participants was estimated by asking them on any background information they had about the app.

Normally, I read the trademark and function of the drug on this app, like whether it controls sugar or blood pressure, as well as when and how many drugs I take.

(User6_22_24_26)

I input my medication information and set it when to remind me. Then it will notify me and jump out at that time to ask if I have taken my medicine, and then I will answer whether I have taken it or not by pressing the button “taken”, but I don't know what would happen if I press “have not taken”.

(User10_6_12)

Participants were able to describe the mechanisms of use, functions, and most likely purposes of the app. Most participants also mentioned the developers of this app and how they knew about this app.

### 3.2 Theme 2: User Perception of Evidence Presented in Support of the Drug Adherence‐Enhancing App

Users were asked about the information they knew and how it influenced their opinions about the app. Some interviewees mentioned encountering information online, but they didn't believe it was crucial for forming their own conclusions.

I have probably read most of them when surfing the Internet and the majority of them introduced this kind of application, reminding people to take medicine.

(User2_26_28_30)

They won't affect my attitude. It doesn't matter if I hear about nothing.

(User2_32)

I haven't seen any reports or papers about this so far.

(User12_18_20)

Surprisingly, many participants admitted to not reading any evidence at all.

### 3.3 Theme 3: User Preferences Toward the Electronic App

The users' perception and attitudes towards the app were captured to understand their preferences regarding the drug‐adherence enhancing app. Many interviewees expressed a positive user experience, as the app's features appeared to be customized to their requirements.

I think it is positive as it can strengthen my initiative in taking medication. Now I take medication according to my schedule every day.

(User8_42_44)

The bell rings and vibrates well. I will remember to take the medicine promptly.

(User3_46)

I think the process of downloading from the beginning, to inputting drugs and other functions is very simple.

(User10_63_65)

Good. It is clear to see what medicine to take in the morning and what to take in the evening.

(User1_67_69)

These features included auditory and vibration alerts, the ability to download and import medication details, and other practical functions. Additionally, the app provided medication data such as drug names, functions, and personal schedules. The design elements of the app, including the ringtone and font, were also well‐received. In comparison to other similar apps, the majority of interviewees indicated a preference for “My eDrug Manager.”

### 3.4 Theme 4: Perceived Difficulty of Using the Electronic App

When users were asked about issues they encountered while using the app, several problems were highlighted. While others felt that the app's functions were too simplistic and lacked features such as setting up medical appointments and accessing health reports. These issues raised concerns about the varying levels of interactivity and customization required by different users, which ultimately had a negative impact on their overall perception of the app.

After I set it up, it kept ringing, and it would ring in the middle of the night to remind me. I once sent a message in WhatsApp to contact for consultation, but it didn't work after they helped me upgrade. And I couldn't contact him later. Some websites also taught me to set up but it was not useful.

(User10_18_20_22_24)

These included connectivity issues and a lack of flexibility in adjusting reminder frequency, timings, and volume. Some users expressed dissatisfaction with the lack of continuous and helpful technical support.

Frequent pop‐up “error” and “fail to connect”.

(User8_28_154_156)

The performance of this Android phone is not that good. It would be better if both Android and iPhone can be used to perform the apps' function well.

(User9_34_40_41)

### 3.5 Theme 5: Adaptability and Trialability of the Electronic App

Participants were asked about their opinions regarding the adaptability of the app to meet local needs and reach potential users. While there was a general consensus on the feasibility of adaptation, some participants believed that additional assistance might be necessary.

Compared to traditional methods such as using paper or computers to record, apps are more convenient and accurate. Because everyone has a phone and it's easier to handle.

(User8_77_74_76)

I think this app can be used by all patients, including elderly people, as long as they can use their mobile phones.

(User9_44_45_46)

It depends on the patient's own situation. If he is still mentally active, there is no problem; If he has a poor condition, he may need some help from family.

(User6_285)

Several interviewees expressed a desire for the app to be more feasible for chronic disease and elderly cases, as they are more likely to experience confusion and forgetfulness regarding medication doses due to long‐term, stable medication routines. Some participants acknowledged that the app could pose challenges for the elderly, depending on their level of mental capability. Recommendations were made to add specific supports, such as louder alerts, to accommodate specific disabilities. However, it was also mentioned by some interviewees that the current versions of the app were not suitable for disabled patients, particularly those who are deaf.

### 3.6 Theme 6: Suggestions for Improvement

When asked about the changes or alterations they believed would be necessary for the app to work effectively, users provided several suggestions.

It would be better if you could add messages or WhatsApp reminder after the application rings. Manual confirmation can be added to double confirm that you really took the medicine.

(User3_50_51_70)

You can cooperate with HA to help change the medication, and it can actively put the medication on the application, making it less troublesome for users.

(User12_88)

The function of this app is often more like a memo, just to help you check when to take the medicine, but there is no explanation for the efficacy or how to take the medicine. I think it's better to add a consultation function to provide more support.

(User6_73)

Many proposed different styles of reminders, such as reminder messages, audio prompts with phrases like “Time for medication!”, manual confirmations, and visual or light‐based indicators. Efficient technical support was also emphasized, as users recognized the varying levels of proficiency among app users and the importance of receiving timely assistance for the app's success. Other suggestions included incorporating comprehensive functions like regular health reports, allowing users to easily monitor their health and medication progress. Additionally, users recommended integrating the app with hospitals to reduce duplicate records and manual inconsistencies.

### 3.7 Theme 7: Effectiveness of the Electronic App

Participants were asked about any changes in medication, blood pressure, or their attitude towards the effectiveness of “My eDrug Manager” app.

It can help me establish the habit of recording my blood pressure, so it helps me control my blood pressure.

(User8_251_253)

Because it's easy to operate, you'll feel it is beneficial, and it has already integrated into your life.

(User8_246)

As I take the medicine on time every day, my physical condition is stable.

(User8_250)

I will recommend others to try it, because sometimes some people really don't remember to take their medicine, and in severe cases, they take it every few days. It is recommended that anyone who needs to take medicine to give it a try to see if they can help.

(User4_100)

Participants reported no changes in medication or blood pressure, indicating stable health conditions. They held a positive attitude towards the “My eDrug Manager” app, finding it useful for enhancing drug adherence. They believed it was useful for enhancing drug adherence and were inclined to recommend it to their family and friends. Some mentioned it helped improve living habits by reminding them to take medication and record blood pressure every day.

## 4 Discussion

The study found users reporting the eHealth app to be helpful with monitoring medication intake and blood pressure, ultimately effective in boosting medication adherence. These findings align with previous literature on the effectiveness of smartphone apps in managing hypertension [9, 13, 14]. The app demonstrated good usability, adaptability, and trialability. The researcher discovered the seven themes through inductive thematic analysis of interview transcripts. The qualitative data from the study revealed significant insights across seven themes, providing valuable considerations for future implementation efforts and potential upgrades. The eHealth app effectively increases medication adherence through tailored reminders and improved health literacy, leading to positive lifestyle changes, enhanced user engagement, and overall better health outcomes compared to non‐use.

Participants' positive perceptions of the app stemmed from three key aspects. Firstly, the reminder functions of the app were well‐designed, with suitable auditory and vibrating alerts, tailored to enhance drug adherence. This finding aligns with another qualitative study, which found that patients preferred having a variety of content delivery formats to choose from [15]. Secondly, the provision of medication information, including names, functions, and schedules, allowed users to better understand their medications and schedules, thereby improving their health literacy.

Similar research has shown that integrating personalized health information technologies can effectively improve medication adherence in diabetes management interventions [16]. Lastly, the simple interface for downloading information, importing medication details, and overall functional usage was beneficial to most users. A straightforward user interface is particularly important for elderly users with limited technological abilities, especially those who have hypertension and are expected to use the app regularly. Other studies have also recommended adopting user‐centered approaches when developing apps to improve medication adherence [17]. Overall, the study highlights the positive impact of the app on medication adherence and provides valuable insights for future development and implementation efforts.

Major perceived difficulties were associated with app usage and technical problems of customizing reminder frequency, connection issues, and compatibility problems across different mobile phone systems. Participants reported struggles in accessing customer service or technical support, suggesting a hotline for immediate feedback on technical issues. The streamlined utility of the app has been shown to directly impact user experience, hence its of great importance to eHealth interventions such as the app in this study [18]. Literature supports the importance of effective and timely technical support for health apps, as many apps suffer from poor usability, making them difficult to use [19].

Interventional efforts to improve patient drug adherence have been extensively studied by applying modification strategies broadly categorized as behavioral, educational, or organizational [11, 20, 21, 22]. Behavioural interventions are assisted by tools such as alarm devices, reminder lists, pill organizers, monitoring feedback systems, group support, and follow‐up visits to modify the patient's surroundings [23]. Educational interventions empower patients by providing knowledge about their medications, their impact on health status, and the importance of adherence to facilitate informed decision making. However, these conventional approaches lack patient participation, self‐management opportunities, and access to information [11, 20, 21, 22, 23, 24, 25].

Qualitative data revealed mixed findings among interviewees regarding reminder settings and the overall function of the app. Some participants expressed preferences for different reminder features, such as volume and types of rings. Suggestions were made to have louder ringtones for elderly users, emphasizing the importance of customized settings. These findings align with previous studies that have shown the effectiveness of personalized tools [26]. For instance, providing personalized content such as medication information and tips for diet and exercise tailored to each individual's specific medication can enhance adherence. A cluster randomized controlled trial involving 270 participants focused on hypertension control and showed that improving patient knowledge about medications can lead to improved adherence [27, 28].

Apart from that, some users wanted more comprehensive functions like medical appointments, while others enjoyed the simple function that was specifically designed to remind medication taking. It is difficult to cater to all tastes. Therefore, if this app can be launched in two versions, it may meet the needs of different people, though it may not be cost‐effective as it will incur another concern about cost. Some users expressed a desire for more comprehensive functions, such as including medical appointments, while others appreciated the simplicity of the app, designed specifically for medication reminders. It is challenging to cater to everyone's preferences.

Launching two versions of the app may help meet the diverse needs of different individuals, although it may not be cost‐effective and raises concerns about additional costs. However, non‐adherence‐associated medical complications, as reported by a systematic review, resulted in 4% of all hospital admissions and 29% of all medication‐related problems, with majority of the cases considered preventable with adherence [29]. In the United States, suboptimal adherence has been estimated to account for up to 10% of total healthcare costs [30].

Another limitation of the app is the lack of interactivity. Interviewees expressed the need for more feedback, such as regular personal health reports that reflect their medication intake progress and changes in health indicators. Empirical research has highlighted the importance of interactivity for e‐health engagement, as it allows users to have a clearer understanding of the information delivered in a more personalized manner and fosters a connection between users and the program [31, 32, 33]. Providing tailored feedback can empower users to take a more active role in managing their health, leading to further behavioral changes.

Some users expressed concerns about the difficulty of confirming whether they actually took their medication or simply pressed “yes” on the app. While suggestions were made to implement requirements like taking a photo or manual double confirmation, these could increase user burden, reduce user experience, and deviate from the intended purpose of the app [34]. Ultimately, the primary goal of this electronic app is to empower patients to improve adherence and modify medication‐taking behavior. Interestingly, many users reported changes in their lifestyle habits after using the app. However, measures should be implemented to promote intention formation, improve the accuracy of response rates, and ensure high levels of engagement [35]. Incorporating a self‐monitoring feature along with personalized goal setting, tailored performance feedback, and follow‐up checks may be effective in addressing inaccurate reports. Additionally, providing manual support for individuals who have not taken their medication for an extended period is necessary.

Users mentioned the lack of interconnectivity with the hospital system as another perceived difficulty. They had to manually input their medication data into the app, and whenever there was a change in their prescription, they had to update the medication information in the app. This inconvenience was also noted in a study that examined 160 adherence apps, highlighting the need for manual data entry on users' phones. [32] To address this limitation, establishing a connection between the app and the hospital system would be beneficial. This would allow for automatic reading and uploading of prescriptions from the hospital to the app. Moreover, it would enable the sharing of health data between both sides, facilitating remote communication between hospitals and patients. By achieving this interconnection, the problem of manual data entry and the need for constant updates could be resolved.

This study reveals an enhancement on the medication adherence of elderly hypertensive patients who are not adherent to their prescribed medications. Mobile Health (mHealth) interventions have been suggested as an effective measure in assisting patients' self‐management of chronic disease, which included medication reminder, refilling of drugs, monitoring of drug‐taking behaviour, and the interaction between the physician and patients. On the other hand, poor adherence or non‐adherence is strongly associated with medication‐related hospitalizations, morbidity and mortality rates, as well as a substantial health care burden. Therefore, in the long run, the mobile applications could have significant implications for health outcomes and health care costs.

This qualitative study had several strengths. It captured the in‐depth meaning and implications of individual narratives, providing rich insights. Use of CFIR added pragmatism and guided the assessment of contextual determinants of implementation, making it widely applicable [36]. Another strength was the long duration of app usage (12 months), allowing for a deeper exploration of participants' perspectives.

However, there are limitations to be acknowledged. Firstly, the rapid pace of technological development requires frequent updates to keep the findings relevant. Secondly, the modest sample size and purposive sampling strategy, which included only three subgroups of patients, limit the generalizability of the findings. Future multi‐center studies should involve diverse population groups, including ethnic minorities and individuals with different chronic conditions, to enhance diversity and generalizability. Additionally, conducting some interviews via phone limited the observation of body language and personal contact. However, there is evidence suggesting that the absence of eye contact can facilitate criticism of the app and encourage disclosure of sensitive information [37, 38].

## 5 Conclusion

In conclusion, this qualitative study highlighted positive aspects of a medication adherence‐enhancing app for patients with chronic diseases. However, there is room for improvement to enhance user‐friendliness. Furthermore, the qualitative study only included opinions from small portion of users which may not be able to summarize overall comments about the apps. Future research should consider prospective randomized trials to evaluate the app's effectiveness in improving disease control and medication adherence among individuals with suboptimal compliance.

## Author Contributions

**Sichen Wang:** conceptualization, supervision, writing – original draft, formal analysis, and data curation. **Junjie Huang:** conceptualization, supervision, writing – original draft, formal analysis, and data curation. **Apurva Sawhney:** writing – original draft. **Xianjing Liu:** writing – review and editing. **Chaoying Zhong:** writing – review and editing. **Jianli Lin:** writing – review and editing. **Junjie Hang:** writing – review and editing. **Claire Chenwen Zhong:** writing – review and editing. **Jinqiu Yuan:** writing – review and editing. **Martin C.S. Wong:** writing – review and editing, conceptualization, and supervision.

## Ethics Statement

Ethics application was approved by The Joint Chinese University of Hong Kong—New Territories East Cluster (CUHK‐NTEC) Clinical Research Ethics Committee; reference no. 2018.650.

## Consent

Patients signed informed consent forms and reminded by moderator before interview regarding the voluntary nature of this study and their rights to withdraw from the study at any moment.

## Conflicts of Interest

The authors declare no conflicts of interest.

## Transparency Statement

The authors Martin C.S. Wong, Junjie Huang, and Sichen Wang affirm that this manuscript is an honest, accurate, and transparent account of the study being reported; that no important aspects of the study have been omitted; and that any discrepancies from the study as planned (and, if relevant, registered) have been explained. All authors have read and approved the final version of the manuscript. Martin C.S. Wong had full access to all of the data in this study and takes complete responsibility for the integrity of the data and the accuracy of the data analysis.

## Supporting information

Supplementary document eHealth‐HTAdherence.

## Data Availability

Data is available upon request from the corresponding author.

## References

[1] “Adherence to Long‐Term Therapies: Evidence for Action,” World Health Organization, published 2023, https://www.paho.org/en/documents/who-adherence-long-term-therapies-evidence-action-2003.

[2] L. Osterberg and T. Blaschke , “Adherence to Medication,” New England Journal of Medicine 353, no. 5 (August 2005): 487–497, 10.1056/NEJMra050100. 16079372

[3] C. Franchi , I. Ardoino , M. Ludergnani , G. Cukay , L. Merlino , and A. Nobili , “Medication Adherence in Community‐Dwelling Older People Exposed to Chronic Polypharmacy,” Journal of Epidemiology and Community Health 75, no. 9 (September 2021): 854–859, 10.1136/jech-2020-214238. 33500324

[4] P. S. Nishtala and M. S. Salahudeen , “Temporal Trends in Polypharmacy and Hyperpolypharmacy in Older New Zealanders Over a 9‐Year Period: 2005–2013,” Gerontology 61, no. 3 (2015): 195–202, 10.1159/000368191. 25428287

[5] M. Burnier and B. M. Egan , “Adherence in Hypertension,” Circulation Research 124, no. 7 (March 2019): 1124–1140, 10.1161/CIRCRESAHA.118.313220. 30920917

[6] “Report of Population Health Survey 2014/15, vol. 2023,” Surveillance and Epidemiology Branch, published December 14, 2017, https://www.chp.gov.hk/en/static/51256.html.

[7] “Hong Kong Reference Framework for Hypertension Care for Adults in Primary Care Settings (Patient Version),” Primary Care Office DoH, published July 2022, https://www.healthbureau.gov.hk/pho/files/e_hypertension_care_patient.pdf.

[8] “Mobile Technology Fact Sheet,” Pew Research Center, published April 7, 2021, https://www.pewresearch.org/internet/fact-sheet/mobile/.

[9] I. Ahmed , N. S. Ahmad , S. Ali , et al., “Medication Adherence Apps: Review and Content Analysis,” JMIR mHealth and uHealth 6, no. 3 (March 2018): e62, 10.2196/mhealth.6432. 29549075 PMC5878368

[10] M. Vervloet , A. J. Linn , J. C. M. van Weert , D. H. de Bakker , M. L. Bouvy , and L. van Dijk , “The Effectiveness of Interventions Using Electronic Reminders to Improve Adherence to Chronic Medication: A Systematic Review of the Literature,” Journal of the American Medical Informatics Association 19, no. 5 (September/October 2012): 696–704, 10.1136/amiajnl-2011-000748. 22534082 PMC3422829

[11] T. D. Aungst , “Smartphone Medication Adherence Apps: Potential Benefits to Patients and Providers: Response to Dayer et al.,” Journal of the American Pharmacists Association 53, no. 4 (July/August 2013): 344–345, 10.1331/JAPhA.2013.13102. 23892800

[12] B. C. O'Brien , I. B. Harris , T. J. Beckman , D. A. Reed , and D. A. Cook , “Standards for Reporting Qualitative Research: A Synthesis of Recommendations,” Academic Medicine 89, no. 9 (2014): 1245–1251. 24979285 10.1097/ACM.0000000000000388

[13] A. Kassavou , M. Wang , V. Mirzaei , S. Shpendi , and R. Hasan , “The Association Between Smartphone App‐Based Self‐Monitoring of Hypertension‐Related Behaviors and Reductions in High Blood Pressure: Systematic Review and Meta‐Analysis,” JMIR mHealth and uHealth 10, no. 7 (July 2022): e34767, 10.2196/34767. 35819830 PMC9328789

[14] H. Xu and H. Long , “The Effect of Smartphone App‐Based Interventions for Patients With Hypertension: Systematic Review and Meta‐Analysis,” JMIR mHealth and uHealth 8, no. 10 (October 2020): e21759, 10.2196/21759. 33074161 PMC7605981

[15] J. Linardon , T. King , A. Shatte , and M. Fuller‐Tyszkiewicz , “Usability Evaluation of a Cognitive‐Behavioral App‐Based Intervention for Binge Eating and Related Psychopathology: A Qualitative Study,” Behavior Modification 46, no. 5 (September 2022): 1002–1020, 10.1177/01454455211021764. 34075803

[16] D. Tao , T. Wang , T. Wang , S. Liu , and X. Qu , “Effects of Consumer‐Oriented Health Information Technologies in Diabetes Management Over Time: A Systematic Review and Meta‐Analysis of Randomized Controlled Trials,” Journal of the American Medical Informatics Association 24, no. 5 (September 2017): 1014–1023, 10.1093/jamia/ocx014. 28340030 PMC7651962

[17] E. E. Ali , J. L. Leow , L. Chew , and K. Y. L. Yap , “Patients' Perception of App‐Based Educational and Behavioural Interventions for Enhancing Oral Anticancer Medication Adherence,” Journal of Cancer Education 33, no. 6 (December 2018): 1306–1313, 10.1007/s13187-017-1248-x. 28707206

[18] D. M. Steinberg , M. C. Kay , L. P. Svetkey , et al., “Feasibility of a Digital Health Intervention to Improve Diet Quality Among Women With High Blood Pressure: Randomized Controlled Feasibility Trial,” JMIR mHealth and uHealth 8, no. 12 (December 2020): e17536, 10.2196/17536. 33284116 PMC7752529

[19] J. Torous , J. Nicholas , M. E. Larsen , J. Firth , and H. Christensen , “Clinical Review of User Engagement With Mental Health Smartphone Apps: Evidence, Theory and Improvements,” Evidence Based Mental Health 21, no. 3 (2018): 116–119, 10.1136/eb-2018-102891. 29871870 PMC10270395

[20] R. Brian Haynes , K. Ann McKibbon , and R. Kanani , “Systematic Review of Randomised Trials of Interventions to Assist Patients to Follow Prescriptions for Medications,” Lancet 348, no. 9024 (August 1996): 383–386, 10.1016/s0140-6736(96)01073-2. 8709739

[21] M. M. Graves , M. C. Roberts , M. Rapoff , and A. Boyer , “The Efficacy of Adherence Interventions for Chronically Ill Children: A Meta‐Analytic Review,” Journal of Pediatric Psychology 35, no. 4 (May 2010): 368–382, 10.1093/jpepsy/jsp072. 19710248

[22] V. Kini and P. M. Ho , “Interventions to Improve Medication Adherence: A Review,” Journal of the American Medical Association 320, no. 23 (December 2018): 2461–2473, 10.1001/jama.2018.19271. 30561486

[23] P. Harbig , I. Barat , and E. M. Damsgaard , “Suitability of an Electronic Reminder Device for Measuring Drug Adherence in Elderly Patients With Complex Medication,” Journal of Telemedicine and Telecare 18, no. 6 (2012): 352–356. 22912488 10.1258/jtt.2012.120120

[24] N. C. Wilhelmsen and T. Eriksson , “Medication Adherence Interventions and Outcomes: An Overview of Systematic Reviews,” European Journal of Hospital Pharmacy 26, no. 4 (July 2019): 187–192, 10.1136/ejhpharm-2018-001725. 31338165 PMC6613929

[25] M. Viswanathan , C. E. Golin , C. D. Jones , et al., “Interventions to Improve Adherence to Self‐Administered Medications for Chronic Diseases in the United States,” Annals of Internal Medicine 157, no. 11 (2012): 785–795. 22964778 10.7326/0003-4819-157-11-201212040-00538

[26] F. Yasmin , L. Ali , B. Banu , F. B. Rasul , R. Sauerborn , and A. Souares , “Understanding Patients' Experience Living With Diabetes Type 2 and Effective Disease Management: A Qualitative Study Following a Mobile Health Intervention in Bangladesh,” BMC Health Services Research 20, no. 1 (January 2020): 29, 10.1186/s12913-019-4811-9. 31918704 PMC6953219

[27] A. B. N. Atreja and S. R. Levy , “Strategies to Enhance Patient Adherence: Making It Simple,” Medscape 7, no. 1 (2005): 4. PMC1681370 16369309

[28] Q. Tu , L. D. Xiao , S. Ullah , J. Fuller , and H. Du , “A Transitional Care Intervention for Hypertension Control for Older People With Diabetes: A Cluster Randomized Controlled Trial,” Journal of Advanced Nursing 76, no. 10 (October 2020): 2696–2708, 10.1111/jan.14466. 32744373

[29] P. Mongkhon , D. M. Ashcroft , C. N. Scholfield , and C. Kongkaew , “Hospital Admissions Associated With Medication Non‐Adherence: A Systematic Review of Prospective Observational Studies,” BMJ Quality & Safety 27, no. 11 (November 2018): 902–914, 10.1136/bmjqs-2017-007453. 29666309

[30] M. McGuire and M. J. Iuga , “Adherence and Health Care Costs,” Risk Management and Healthcare Policy 7 (2014): 35–44, 10.2147/RMHP.S19801. 24591853 PMC3934668

[31] A. Barak , B. Klein , and J. G. Proudfoot , “Defining Internet‐Supported Therapeutic Interventions,” Annals of Behavioral Medicine 38, no. 1 (August 2009): 4–17, 10.1007/s12160-009-9130-7. 19787305

[32] L. Dayer , S. Heldenbrand , P. Anderson , P. O. Gubbins , and B. C. Martin , “Smartphone Medication Adherence Apps: Potential Benefits to Patients and Providers,” Journal of the American Pharmacists Association 53, no. 2 (March/April 2013): 172–181, 10.1331/JAPhA.2013.12202. 23571625 PMC3919626

[33] H. de Vries , S. P. J. Kremers , T. Smeets , J. Brug , and K. Eijmael , “The Effectiveness of Tailored Feedback and Action Plans in an Intervention Addressing Multiple Health Behaviors,” American Journal of Health Promotion 22, no. 6 (2008): 417–424, 10.4278/ajhp.22.6.417. 18677882

[34] A. Middelweerd , S. J. Te Velde , J. S. Mollee , M. C. Klein , and J. Brug , “App‐Based Intervention Combining Evidence‐Based Behavior Change Techniques With a Model‐Based Reasoning System to Promote Physical Activity Among Young Adults (Active2Gether): Descriptive Study of the Development and Content,” JMIR Research Protocols 7, no. 12 (December 2018): e185, 10.2196/resprot.7169. 30578198 PMC6320419

[35] S. Michie , C. Abraham , C. Whittington , J. McAteer , and S. Gupta , “Effective Techniques in Healthy Eating and Physical Activity Interventions: A Meta‐Regression,” Health Psychology 28, no. 6 (November 2009): 690–701, 10.1037/a0016136. 19916637

[36] L. J. Damschroder , D. C. Aron , R. E. Keith , S. R. Kirsh , J. A. Alexander , and J. C. Lowery , “Fostering Implementation of Health Services Research Findings Into Practice: A Consolidated Framework for Advancing Implementation Science,” Implementation Science 4 (August 2009): 50, 10.1186/1748-5908-4-50. 19664226 PMC2736161

[37] R. J. Fleischmann , M. Harrer , A. C. Zarski , H. Baumeister , D. Lehr , and D. D. Ebert , “Patients' Experiences in a Guided Internet and App‐Based Stress Intervention for College Students: A Qualitative Study,” Internet Interventions 12 (June 2018): 130–140, 10.1016/j.invent.2017.12.001. 30135777 PMC6096317

[38] P. Carlbring , L. Ekselius , and G. Andersson , “Treatment of Panic Disorder Via the Internet: A Randomized Trial of CBT vs. Applied Relaxation,” Journal of Behavior Therapy and Experimental Psychiatry 34, no. 2 (June 2003): 129–140, 10.1016/s0005-7916(03)00026-0. 12899896

