# Supplementary material for: Evaluation of an Electronic Application for Enhancing Medication Adherence Among Hypertensive Patients: A Qualitative Research Based on Consolidated Framework for Implementation Research (CFIR)
Source: Health Sci Rep. 2025 Apr 29;8(5):e70758. doi: 10.1002/hsr2.70758 (PMC12040757; doi:10.1002/hsr2.70758)
Supplement: Supplementary file 1 — Supplementary document eHealth‐HTAdherence. [file HSR2-8-e70758-s001.pdf]

Supplementary legends

Supplementary Table 1. The characteristics of the subjects

Supplementary Table 1. The characteristics of the subjects

Interviewee	Gender	Age	Age Group	No. of chronic medication	chronic medication	Education level
1	F	70	≥ 65	2	Diabetes, hypertension	Secondary school
2	F	69	≥ 65	2	Overweight, hypertension	Secondary school
3	M	68	≥ 65	2	Overweight, hypertension	Secondary school
4	M	52	40-64	1	hypertension	Tertiary school or above
5	M	63	40-64	2	Overweight, hypertension	Tertiary school or above
6	F	61	40-64	1	hypertension	Uneducated/Preschool/Primary
7	F	62	40-64	3	Diabetes, overweight, hypertension	Tertiary school or above
8	M	60	40-64	3	overweight, hypertension, heart disease	Secondary school
9	M	74	≥ 65	1	hypertension	Tertiary school or above
10	M	61	40-64	3	overweight, hypertension, hyperlipidemia	Tertiary school or above
11	M	75	≥ 65	2	Overweight, hypertension	Secondary school
12	F	65	≥ 65	2	hypertension, heart disease	Secondary school
